# Proteome Remodelling in *Candida auris* During Early Host Adaptation In Vitro and In Vivo

**DOI:** 10.3390/jof12090649

**Published:** 2026-09-01

**Authors:** Rounik Mazumdar, Ana Bjelanovic

**Affiliations:** Center for Medical Biochemistry, Medical University of Vienna, Vienna BioCenter, 1030 Vienna, Austria

**Keywords:** *Candida auris*, host–pathogen, proteomics, in vitro, macrophage, in vivo, mouse, virulence factors, pathogenesis, immune response

## Abstract

*Candida auris* is an emerging fungal pathogen that is posing a serious global health threat due to its high transmissibility and multidrug resistance profile. Despite recent molecular advances in scrutinizing this enigmatic microbe, much of our understanding regarding its pathomechanisms remains unelucidated. Since microbial pathogenesis is modulated by a dynamic interplay between the host and the pathogen, dissecting such host–pathogen interactions involving *C. auris* can shed novel insights into its pathogenic cascade. As such, to characterize the virulence repertoire of *C. auris*, this study applied an integrated quantitative proteomics strategy to scrutinize the early phase of infection. In vitro and in vivo experimental setups based on macrophage co-culture and murine intraperitoneal infection were utilized. Integrated proteomic analysis revealed a coordinated remodelling of cellular processes by *C. auris* during the early host–pathogen interaction phase, including downregulation of translational machinery, modulation of molecules involved in metabolic rewiring, stress responses, and structural rearrangements. Several proteins associated with oxidative stress adaptation, alternative carbon metabolism, and cytoskeletal regulation were differentially abundant during host interactions. Collectively, these findings demonstrate that early adaptation of *C. auris* to host immune pressure involves rapid and context-dependent proteome remodelling that may contribute to fungal survival and persistence during infection.

## 1. Introduction

*Candida auris* (*Candidozyma auris*), a fungal pathogen first identified in 2009 in Japan, has since emerged as a ‘superbug’, proliferating across the globe and causing severe nosocomial outbreaks [1]. Genomic analysis of *C. auris* clinical strains has identified six clades based on geographic locations, including clade I (South Asia), clade II (East Asia), clade III (Africa), clade IV (South America), clade V (Iran), and the very recent clade VI (Singapore), with clade I being the most prevalent [2,3,4]. The pathogen can cause deep-seated infections of the bloodstream (candidemia), wounds, the respiratory and urinary tracts, as well as ear infections, with high mortality rates ranging from 28% to 56% [5,6]. The fungus has been listed by the World Health Organization (WHO) as a ‘critical priority’ pathogen in its fungal priority pathogens list (FPPL), warranting urgent attention and research. Despite recent advances in our understanding of the pathomechanisms of *C. auris*, much remains unknown. The pathogen is particularly problematic owing to its high transmissibility, clinical misdiagnosis, and drug resistance profile, often displaying multidrug resistance (MDR). It has been reported that 90% of *C. auris* clinical isolates are resistant to fluconazole, 35% to amphotericin B (AmB), 7% to echinocandins, 3% to flucytosine, and over 40% were reported to be resistant to more than two classes of antifungals [7]. Treatment options can therefore be limited by acquired resistance and by the availability of a low number of antifungal families. Understandably, current research has largely focused on the antifungal resistance mechanisms of *C. auris* [8,9]. However, to effectively combat the pathogen, a rigorous understanding of its virulence cascade is also warranted. Emerging reports are pointing towards a very complex pathogenic cascade harboured by the microbe [10]. Evidence suggests that *C. auris* can efficiently adapt to the host microenvironment in order to evade the host–immune system and establish and maintain an infection [10]. Henceforth, concurrent with the ongoing focus on addressing drug resistance of *C. auris*, the virulence aspect of the pathogen also needs to be tackled. As such, this study focuses on the molecular basis of virulence in *C. auris,* aiming to shed novel insights into its infection biology. To address this, a quantitative proteomics strategy was applied to identify the virulence factors modulating host–pathogen interactions during the early phase of infection. Infection models using in vitro co-culture setups with immune cells and an in vivo murine infection model were established. Following a brief exposure of *C. auris* to host cells both in vitro and in vivo, re-isolated fungal cells were subjected to quantitative proteomics analysis to identify the molecules mediating host–pathogen interactions during the initial stages of infection.

## 2. Materials and Methods

### 2.1. Fungal Culture

In this study, the *C. auris* isolate 1133/P/13-R (clade I, origin Vallabhbhai Patel Chest Institute; VPCI) was used, owing to its multidrug-resistant phenotype against amphotericin B and fluconazole [11]. Fungal cells were routinely cultured in YPD liquid medium (1% yeast extract, 2% peptone, and 2% glucose) at 30 °C with constant shaking at 200 rpm.

### 2.2. Ethical Statement

All animal experiments were conducted in accordance with the approval received from the Ethics Committee of the Medical University of Vienna and the Austrian Federal Ministry of Science and Research (BMBWF-66.009/0436-V/3b/2019), adhering to European legislation for animal experimentation [12].

### 2.3. Isolation of Primary Macrophages and In Vitro Co-Culture

Isolation and cultivation of primary bone marrow-derived macrophages (BMDM) was performed as described previously [13]. Briefly, after sacrificing 8–12-week-old female C57BL/6 wild-type mice (*Mus musculus*) by cervical dislocation, the abdomen and the hind legs were sterilized with 70% ethanol. This was followed by isolation of the femurs and tibias, after which the bones were briefly rinsed with 70% ethanol and stored in 25 mL of phosphate-buffered saline (PBS) before moving on to the next mouse. After all bones were collected from mice and stored in PBS, they were aseptically separated at the knee joints. After this, the bones were transferred into a sterile mortar and pestle with 10 mL of cold Dulbecco’s Modified Eagle Medium (DMEM) (Merck, Darmstadt, Germany) for grinding. After thorough grinding, the entire suspension was passed through a 0.22 µm cell strainer. Cells were then harvested at 300 g for 5 min at 4 °C and incubated in red cell lysis buffer in a ratio of 500 µL/leg for 2 min at room temperature. Afterwards, the reaction was stopped by the addition of 25 mL of cold DMEM. Bone marrow cells were harvested at 300 g for 5 min at 4 °C and resuspended in L929 murine fibroblast cell (ATCC)-conditioned medium (BMDM medium) for differentiation and cell culture. For co-culture assays, macrophages were counted by a CASY counter (Roche, Basel, Switzerland) and seeded in 6-well plates with 1 × 10^5^ cells/well at a multiplicity of infection (MOI) of 10:1; fungi:macrophage and incubated in 2.5 mL of DMEM at 37 °C in a 5% CO_2_ incubator for 2 h. The control cultures contained no immune cells. Experiments were performed as biological triplicates with three co-cultures per replicate. Additionally, macrophage fungal kill efficiency was monitored at an MOI of 1:1. Following 2 h of incubation, 500 µL of 0.2% Triton X-100 was added to each well, after which the cells were gently scraped from the wells using fresh cell scrapers. Then, the suspensions were transferred into 15 mL Falcon tubes, followed by 30 s of vortexing. An aliquot of this was utilized for plating on YPD agar plates for Colony-Forming Unit (CFU) determination with appropriate dilutions. The cell suspensions in 15 mL Falcon tubes were then centrifuged at 3000 *g* for 5 min, followed by 3× washes in sterile ice-cold PBS, with the final cell suspension being processed for proteome isolation. Of note, the 2 h time point was selected to explore the immediate early-phase host–pathogen interaction in contrast to later late-stage invasive processes.

### 2.4. Mouse Infection and Re-Isolation of Fungal Cells from Lavage

In order to investigate the immediate immune response and early host–pathogen interaction, intraperitoneal infection in immunocompetent mice was performed as previously described with slight modifications [14]. Although the intraperitoneal infection model does not fully recapitulate disseminated candidiasis, it provides a well-controlled system for investigating immediate host–pathogen interactions and recovering sufficient fungal biomass for quantitative proteomic analysis. In brief, 6–8-week-old female C57BL/6 mice were injected intraperitoneally with 7.5 × 10^7^ fungal cells suspended in 300 µL of sterile PBS using 27-gauge needles. The control mice received 300 µL of sterile PBS alone. Experiments were performed in triplicate with three independent mice per replicate, totalling 9 mice. To enable assessment of rapid proteomic alterations upon initial fungal exposure to the host, mice were sacrificed at 2 h post-infection, and peritoneal lavage was performed by washing the peritoneal cavity three times with sterile PBS. Lavage fluids were pooled to a final 30 mL total volume per animal in sterile 50 mL Falcon tubes. An aliquot of this was utilized for plating on YPD plates for CFU determination with appropriate dilutions. Subsequently, lavage fluids were centrifuged at 300 *g* for 5 min to separate the cellular material from the supernatant. Following this, the supernatant fraction was then further centrifuged at 3000 *g* for 10 min at 4 °C to pellet fungal cells and any residual cellular material. After this, the majority of the supernatant was carefully pipetted out using 10 mL and 1 mL tips, leaving behind approximately 200 µL, undisturbed and retained just above the visible pellet at the bottom of the Falcon tube. From this 200 µL, an aliquot of 10 µL was utilized for visual examination by light microscopy (20×–40× magnification) to confirm the presence of fungal cells. Following this, 300 µL of fungal lysis buffer was added to the 200 µL pellet sample. After which, the total volume of a 500 µL sample containing re-isolated fungal cells was then processed for proteome isolation.

### 2.5. Proteome Isolation

**Figure 1:**
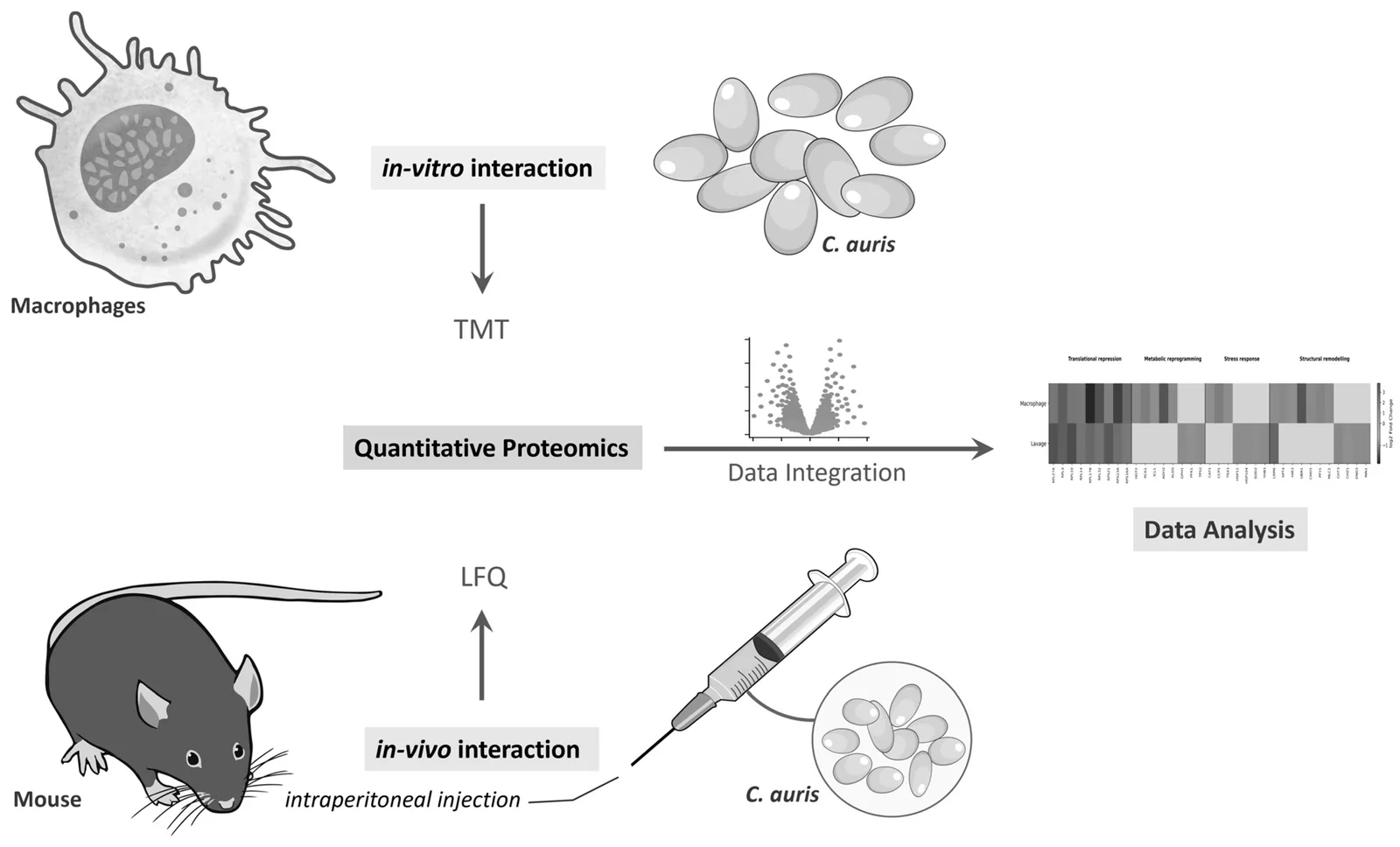
Experimental workflow applied to investigate *C. auris* early-phase host–pathogen interaction.

Following the fungal interactions in vitro and in vivo (Figure 1), protein was isolated from the fungal cells. Briefly, *C. auris* cells were centrifuged at 3000 *g* for 5 min, followed by ice-cold PBS washing 3×, after which the cell pellet was resuspended in 500 μL of ice-cold fungal lysis buffer (1% sodium deoxycholate (SDC), 100 mM Tris-HCl, 150 mM NaCl, 1 mM PMSF, 1 mM EDTA, and 1× cOmplete™ Protease Inhibitor (Roche, Basel, Switzerland)) in 1.5 mL screw-cap tubes. Next, the fungal cells were subjected to mechanical disruption by glass beads using FastPrep™-24 5G Bead Beating (Fisher Scientific, MA, USA). Following this, a small hole was punctured in the bottom of the screw-cap tube using a red-hot flamed needle; the screw-cap tube was then inserted into a 1.5 mL Eppendorf tube and centrifuged at 3000 *g* for 2 min at 4 °C to separate the lysate from the glass beads. The collected lysate in the Eppendorf tube was then subjected to acetone precipitation by adding four volumes of ice-cold 100% acetone, followed by 2 h of incubation at −20 °C. Post-incubation, the samples were centrifuged at 3000 *g* for 5 min to obtain the resulting protein pellet, and the supernatant acetone was discarded. The protein pellet was air-dried on ice and was further processed for mass spectrometric analysis.

### 2.6. Label-Free Quantitative Proteomics of In Vivo Lavage Samples

#### 2.6.1. Sample Preparation for Mass Spectrometry Analysis

The protein pellets were resuspended in 300 µL of 4% (*w*/*v*) SDS, 100 mM Tris/HCl pH 8.5, and incubated at 95° C for 5 min. The lysate was clarified by centrifugation at 16,000 *g* for 10 min at 30° C. The supernatant was transferred to a new tube, and the protein concentration was measured using a 600 nm protein assay kit (Pierce, Waltham, MA, USA) with Sodium dodecyl sulphate (SDS) compatibility reagents. A total of 50 µg protein was reduced by adding 30 µL of 1 M dithiothreitol (DTT) and heating at 95° C for 5 min. Then, it was diluted with 9× 8 M urea in 100 mM Tris/HCl, pH 8.5, transferred to the Filter-Aided Sample Preparation (FASP) filter, and centrifuged for 20 min at 12,000 *g*. Then, it was washed with 200 µL of 8 M urea in 100 mM Tris/HCl, pH 8.5, for 20 min at 12,000 *g*. A total of 100 µL of 50 mM Iodacetamide in 100 mM Tris/HCl, pH 8.5, was added, shaken for 1 min, and incubated for 30 min in the dark at room temperature. Then, it was centrifuged for 10 min at 12,000 *g*, followed by washing twice with 200 µL of 8M urea in 100 mM Tris/HCl, pH 8.5, and three times with 100 µL of 50 mM ammonium bicarbonate (ABC). The filter was transferred to a new collection tube, and 40 µL of 50 mM ABC containing 1 µg of trypsin platinum (Promega, Madison, WI, USA) was added and kept at 37° C overnight. The digested peptides were collected by centrifuging for 15 min at 12,000 *g*. The filter was washed with 40 µL of 50 mM ABC, centrifuged for 15 min at 12,000 *g*, and pooled. The digested peptides were acidified with 10 µL of 10% TFA, and the peptides were desalted using C18 Stagetips [15] and an MCX 96-well plate (Waters, Vienna, Austria).

#### 2.6.2. Liquid Chromatography–Mass Spectrometry Analysis

Peptides were separated on an Ultimate 3000 RSLC nano-flow chromatography system (Thermo-Fisher, MA, USA) using a pre-column for sample loading (Acclaim PepMap C18, 5 mm × 300 µm, 5 μm, Thermo-Fisher) and a C18 analytical column (Acclaim PepMap C18, 50 cm × 75 µm, 2 μm, Thermo-Fisher), applying a segmented linear gradient from 2% to 35% and finally 80% solvent B (80% acetonitrile, 0.1% formic acid; solvent A 0.1% formic acid) at a flow rate of 230 nL/min over 120 min. Eluting peptides were analyzed on an Exploris 480 Orbitrap mass spectrometer (Thermo Fisher) coupled to the column with a FAIMS pro ion source (Thermo-Fisher) using coated emitter tips with the following settings: the mass spectrometer was operated in the data-dependent acquisition (DDA) mode with two FAIMS compensation voltages (CV) set to −45 or −60, with a 1.5 s cycle time per CV. The survey scans were obtained in a mass range of 350–1500 *m*/*z* at a resolution of 60 k at 200 *m*/*z*, with a normalized AGC target at 100%. The most intense ions were selected with an isolation width of 1.2 *m*/*z*, fragmented in the Higher-energy Collisional Dissociation (HCD) cell at 28% collision energy, and the spectra were recorded for a max. 100 ms at a normalized AGC target of 100% and a resolution of 15 k. Peptides with a charge of +2 to +6 were included for fragmentation; the peptide match feature was set to preferred; the exclude isotope feature was enabled; and selected precursors were dynamically excluded from repeated sampling for 45 s.

#### 2.6.3. Data Analysis

Raw data were split into single cv using FreeStyle 1.8 SP2 and processed using the MaxQuant software package (version 1.6.17.0, [16]) and the UniProt *C. auris* reference proteome (version 2021.03, www.uniprot.org), as well as a database of the most common contaminants; mouse proteins identified were explicitly excluded from the data analysis. The search was performed with full trypsin specificity and a maximum of two missed cleavages at a protein and peptide spectrum match false discovery rate of 1%. Carbamidomethylation of cysteine residues was set as fixed, and oxidation of methionine and N-terminal acetylation as variable modifications. For label-free quantification, the “match between runs” feature and the label-free quantification (LFQ) function were activated—all other parameters were left at default. MaxQuant output tables were further processed in R (version 4.6.1) using Cassiopeia_LFQ (https://github.com/maxperutzlabs-ms/Cassiopeia_LFQ, accessed on 20 August 2026). Reverse database identifications, contaminant proteins, protein groups identified only by a modified peptide, protein groups with fewer than two quantitative values in one experimental group, and protein groups with fewer than two razor peptides were removed for further analysis. Missing values were replaced by randomly drawing data points from a normal distribution modelled on the whole dataset (data mean shifted by −1.8 standard deviations; width of distribution of 0.3 standard deviations). Differences between groups were statistically evaluated using the LIMMA package [17] at 5% FDR (Benjamini–Hochberg).

### 2.7. TMT Label-Based Quantitative Proteomics of In Vitro Fungal Cells

#### 2.7.1. Sample Preparation for Mass Spectrometry Analysis

The protein pellets were resuspended in 100 µL of 8M urea and 50 mM ammonium bicarbonate, with sonication and vortexing. The protein was reduced by adding 3.2 µL of 250 mM dithiothreitol (DTT) in 50 mM ammonium bicarbonate and kept at 37° C for 30 min, followed by adding 3.2 µL of 500 mM Iodacetamide in 50 mM ammonium bicarbonate and incubated for 30 min in the dark at room temperature. The alkylation reaction was quenched by adding 1.6 µL of 250 mM DTT for 10 min. The urea concentration was reduced to 4M using 50 mM ABC. Samples were pre-digested for 3 h at 37 °C with Lys-C at an enzyme-to-substrate ratio of 1:50 and subsequently reduced to 1M urea using 50 mM ABC and digested overnight at 37 °C with sequencing-grade trypsin at an enzyme-to-substrate ratio of 1:50. Digests were stopped by acidification with TFA (0.5% final concentration) and desalted on an HLB 96-well plate (Waters, Vienna, Austria). Peptide concentrations were determined and adjusted according to the UV chromatogram peaks obtained with an UltiMate 3000 Dual LC nano-HPLC System (Dionex, Thermo Fisher Scientific, Waltham, MA, USA), equipped with a monolithic C18 column. Desalted samples were concentrated in a SpeedVac concentrator and subsequently lyophilized overnight. Lyophilized peptides were dissolved in 50 μL of 100 mM TEAB. A total of 450 μg of each tandem mass tag (TMT)-10Plex reagent (Thermo Fisher Scientific, MA, USA) was dissolved in 30 μL of 100% anhydrous acetonitrile, and 30 μL was added to the peptide/TEAB mixes at a 1:4 sample:label ratio. The labels used are as follows: 126C: candia_1; 127N: coculture_1; 127C: candia_2; 128N: coculture_2; 128C: candia_3; 129N: coculture_3; 129C: empty; 130N:mouse_1; 130C:mouse_2; and 131:mouse_3. Samples were labelled for 60 min at RT. A total of 0.1 μL of each sample was pooled, mixed with 10 μL of 0.1% TFA, and analyzed by mass spectrometry (MS) to check labelling efficiency. The labelling was repeated twice to make sure all channels had a labelling efficiency of > 98%. For quenching, 8 μL of 5% hydroxylamine was added, and the reaction was incubated for 25 min at RT. Samples were pooled and subsequently desalted with Sep-Pak tC-18 cartridges (Waters, Vienna, Austria). Desalted samples were dried for 30 min in a SpeedVac vacuum centrifuge and subsequently lyophilized overnight. The sample was high pH-fractionated using the Pierce high pH reversed-phase peptide fractionation kit. Nine fractions were collected and lyophilized overnight.

#### 2.7.2. Liquid Chromatography–Mass Spectrometry Analysis

Peptides were separated on an Ultimate 3000 RSLC nano-flow chromatography system (Thermo-Fisher, MA, USA) using a pre-column for sample loading (Acclaim PepMap C18, 5 mm × 300 µm, 5 μm, Thermo-Fisher) and a C18 analytical column (Acclaim PepMap C18, 50 cm × 75 µm, 2 μm, Thermo-Fisher), applying a segmented linear gradient from 2% to 35%, and finally, 80% solvent B (80% acetonitrile, 0.1% formic acid; solvent A 0.1% formic acid) at a flow rate of 230 nL/min over 180 min. Eluting peptides were analyzed on an Orbitrap Eclipse mass spectrometer (Thermo Fisher, MA, USA) coupled to the column with a FAIMS pro ion source (Thermo-Fisher, MA, USA) using coated emitter tips (PepSep, MSWil) with the following settings: The mass spectrometer was operated in DDA mode with three FAIMS compensation voltages (CV) set to −40, −55, or −70, and a 1.2 s cycle time per CV. The survey scans were obtained in a mass range of 375–1500 *m*/*z*, at a resolution of 120 k at 200 *m*/*z*, with a normalized automatic gain control (AGC) target of 100%. The most intense ions were selected with an isolation width of 1.2 *m*/*z*, fragmented in the HCD cell at 30% collision energy, and the spectra were recorded for a max. of 50 ms at a normalized AGC target of 100% in the ion trap. Peptides with a charge of +2 to +6 were included for fragmentation; the peptide match feature was set to preferred; the exclude isotope feature was enabled; and selected precursors were dynamically excluded from repeated sampling for 45 s. High-resolution MS1 survey scans were acquired in the Orbitrap analyzer. Precursors were selected for fragmentation by CID in the linear ion trap (MS2). Real-Time Search was enabled to perform on-the-fly database searching of MS2 spectra using a sample FASTA database, with fixed modifications of carbamidomethyl(C) and TMT10plex (Kn), and variable modifications of oxidation(M) and deamidation (NQ). Only MS2 spectra passing the peptide-level FDR threshold (≤5%) triggered an MS3 event, thereby eliminating TMT quantification of unidentified or co-isolated precursors. MS3 scans utilized 10 (synchronous precursor selection) SPS notches to co-isolate fragment ions for HCD fragmentation and Orbitrap detection of TMT reporter ions (*m*/*z* 110–500).

#### 2.7.3. Data Analysis

Raw data were split into single cv using FreeStyle 1.8 SP2 and were processed using the MaxQuant software package (version 2.0.3.0, [16]) against the UniProt *C. auris* reference proteome (version 2021.03, www.uniprot.org), the UniProt mouse reference proteome (version 2021.03), as well as a database of the most common contaminants. The search was performed in MS3 TMT10 reporter ion mode with isobaric label correction, with full trypsin specificity and a maximum of two missed cleavages at a protein and peptide spectrum match false discovery rate of 1%. Carbamidomethylation of cysteine residues was set as fixed, and oxidation of methionine and N-terminal acetylation as variable modifications. All other parameters were left at default. The MaxQuant output proteinGroups table was further processed in R using Cassiopeia_LFQ (https://github.com/maxperutzlabs-ms/Cassiopeia_LFQ, accessed on 20 August 2026). Only *Candida* samples and mix samples were included in the current manuscript. Reverse database identifications, contaminant proteins, protein groups identified only by a modified peptide, protein groups with fewer than two quantitative values in one experimental group, and protein groups with fewer than 2 razor peptides were removed for further analysis. The protein intensities were median-normalized using only fungal protein intensities. Missing values were replaced by randomly drawing data points from a normal distribution modelled on the whole dataset (the data mean shifted by −1.8 standard deviations, width of distribution of 0.3 standard deviations). Differences between groups were statistically evaluated using the LIMMA package [17] at 5% FDR (Benjamini–Hochberg). To assess potential host peptide interference, an in silico tryptic digest of the *C. auris* and *M. musculus* reference proteomes was performed. Quantified peptides were classified as species-specific or potentially cross-species based on their sequence identity. For proteins containing shared peptides, the numbers of species-specific and cross-species peptides were determined so as to evaluate the potential influence of host-derived peptides on protein quantification.

### 2.8. Proteomics Data Deposition

The mass spectrometry proteomics data have been deposited in the ProteomeXchange Consortium via the PRIDE partner repository [18] with the dataset identifier PXD074745 for label-free proteomics and PXD074762 for TMT-labelled proteomics.

## 3. Results

### 3.1. Interaction of C. auris with Macrophages In Vitro and In Vivo Mice

**Figure 2:**
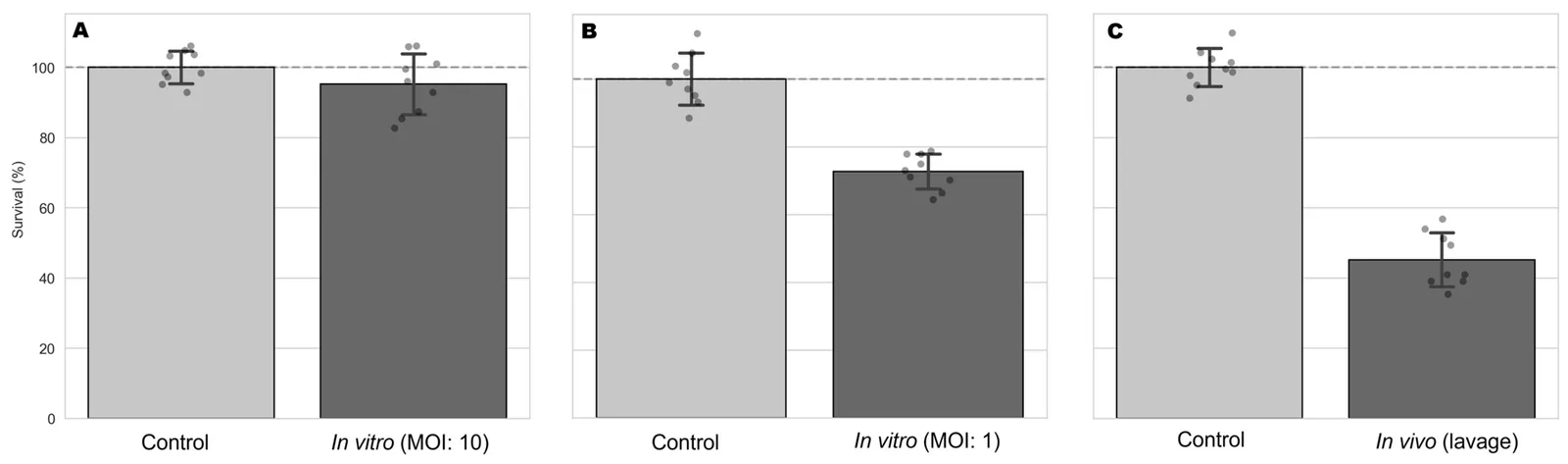
Early-phase kill kinetics of *C. auris* were assessed during in vitro and in vivo conditions with 2 h incubation. Each assay was performed independently and compared with corresponding control groups. (**A**) *C. auris* survival during its interaction with macrophages at an MOI of 10:1 (fungi:macrophage); no significant difference in survival was observed compared to the control (*p* = 0.449). (**B**) *C. auris* killing during its interaction with macrophages at an MOI of 1:1; significant killing was observed (*p* < 0.001). (**C**) *C. auris* survival in vivo; significant killing of fungal cells compared to the control (*p* < 0.001). Statistical significance between the control and experimental groups was determined using an unpaired two-tailed *t*-test.

To determine the susceptibility of *C. auris* to immune-mediated killing during the initial stages of infection, fungal kill kinetics at 2 h under both in vitro and in vivo interaction conditions were evaluated. During the interaction with macrophages in vitro at an MOI of 1:1, *C. auris* was susceptible to immune killing, reflecting the efficiency of macrophages (Figure 2B). However, with an MOI of 10:1 fungal cells, *C. auris* persisted, with no significant killing by immune cells (Figure 2A), indicating that numerical fungal burden influences the kill kinetics of immune cells. In contrast, during in vivo conditions, a significant *C. auris* reduction was observed (Figure 2C), which is consistent with fungal clearance, although contributions from other in vivo factors affecting fungal recovery cannot be excluded.

### 3.2. Proteome Landscape of C. auris During In Vitro and In Vivo Interactions

**Figure 3:**
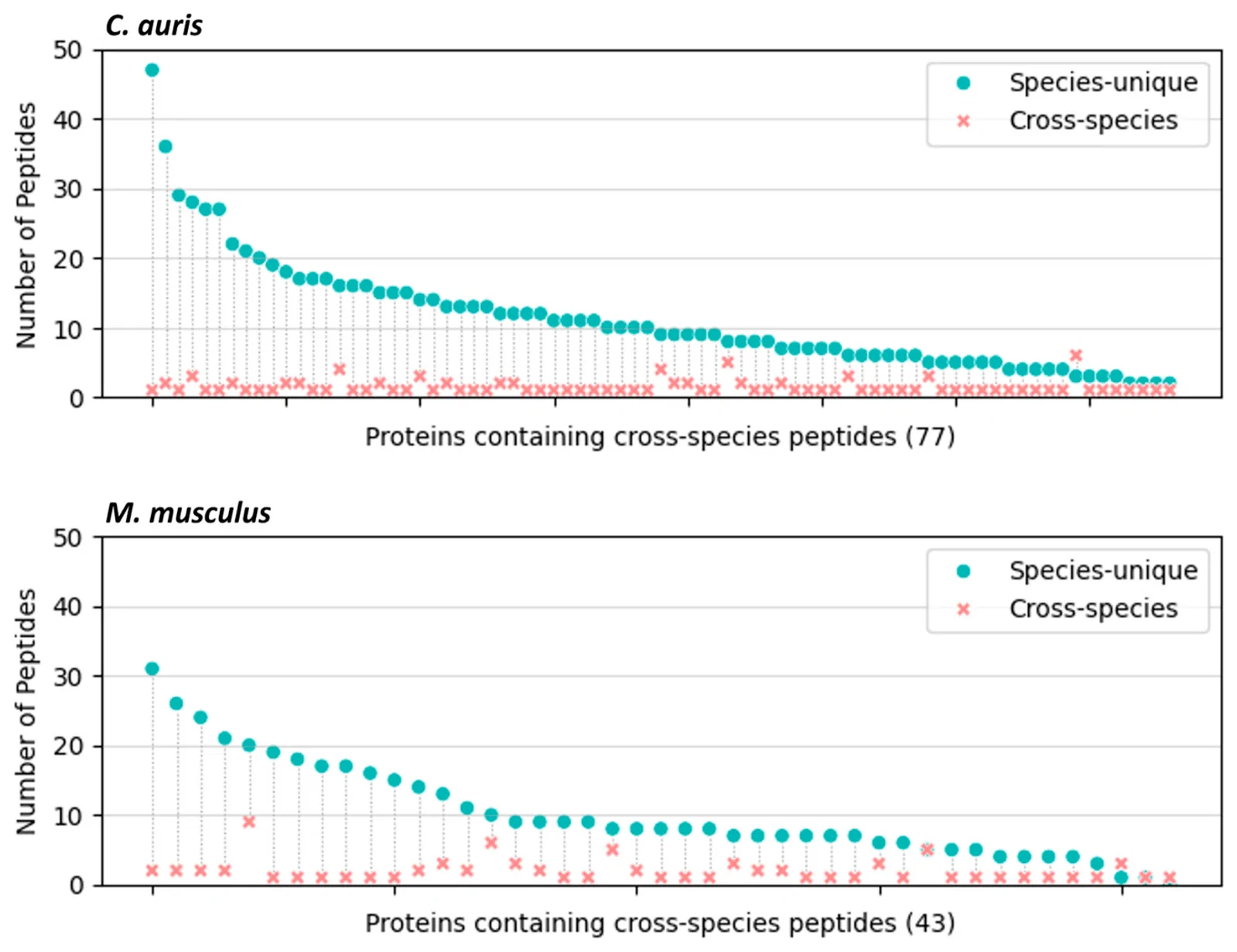
Evaluation of host peptide interference in the fungal proteomic dataset. Quantified peptides were classified as species-specific or potential cross-species peptides using an in silico tryptic digest of the *C. auris* and mouse reference proteomes. Only 196 of 29,171 quantified peptides (0.67%) were identified as potential cross-species peptides, corresponding to 77 of 2979 quantified *C. auris* proteins (2.6%). Species-specific peptides predominated for nearly all affected proteins.

**Figure 4:**
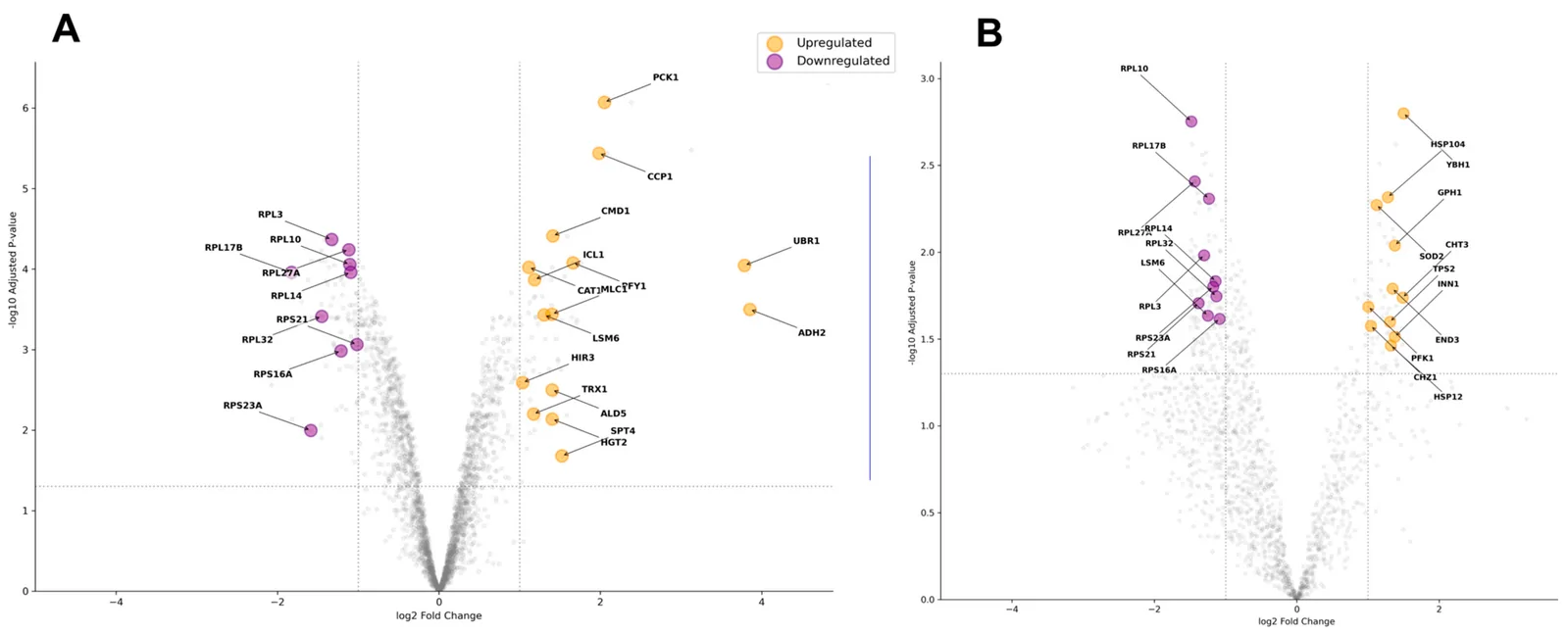
Volcano plots showing proteomic responses of *C. auris* during in vitro macrophage and in vivo lavage interactions when compared to uninfected controls. (**A**) Differentially abundant proteins (DAPs) during *C. auris* interaction with macrophages in vitro. A total of 3034 fungal proteins were identified, with 1073 significant DAPs (adj. *p*.val ≤ 0.05), where 67 proteins were upregulated and 82 were downregulated (log_2_ FC ± 1). (**B**) DAPs of *C. auris* upon in vivo interaction in mice. A total of 2484 *C. auris* proteins were identified, with 272 significant DAPs (adj. *p*.val ≤ 0.05), where 53 proteins were upregulated and 135 proteins were downregulated (log_2_ FC ± 1).

To characterize the proteome landscape of *C. auris* during its interaction with the host, a quantitative proteomics strategy was applied to re-isolated fungal cells upon their interaction with macrophages in vitro and in vivo in mice. To evaluate the potential impact of host contamination on fungal protein quantification, an in silico peptide comparison between the *C. auris* and mouse proteomes was performed, and quantified peptides were classified as species-specific or potential cross-species peptides. Among 29,171 quantified peptides, only 196 (0.67%) were identified as potential cross-species peptides. These peptides were associated with 77 of the 2979 quantified *C. auris* proteins (2.6%). For nearly all affected proteins, species-specific peptides substantially outnumbered shared peptides, indicating that cross-species peptide interference had a minimal impact on protein quantification (Figure 3). To evaluate the potential impact of missing-value imputation on downstream statistical analysis, the distribution of missing values was assessed across the TMT dataset. Of the 2528 *C. auris* proteins subjected to statistical analysis, only seven proteins (0.27%) contained missing values in any sample. None of these proteins were significantly differentially abundant or exhibited large fold changes, indicating that missing-value imputation had a minimal impact on the differential abundance analysis. After exclusion of mouse proteins, a total of 3034 fungal proteins were detected upon in vitro interaction with macrophages (Supplementary Table S1). Of these, 1073 proteins were significantly differentially abundant (adjusted *p*-value (adj. *p*.val) < 0.05), reflecting alterations in the fungal proteome response. Among these differentially abundant proteins (DAPs), 67 proteins were upregulated, while 82 proteins were downregulated (log_2_ fold-change (FC) threshold ± 1) (Figure 4A). The proteome landscape of *C. auris* during the in vivo interaction revealed a total of 2484 proteins, with 272 DAPs (adj. *p*.val < 0.05), where 53 proteins were upregulated and 135 proteins were downregulated (log_2_ FC threshold ± 1) (Figure 4B). Together, these findings demonstrate extensive proteome remodelling by *C. auris* during both in vitro macrophage interaction and in vivo host exposure.

### 3.3. Functional Categorization of Selected Differentially Abundant Proteins

**Figure 5:**
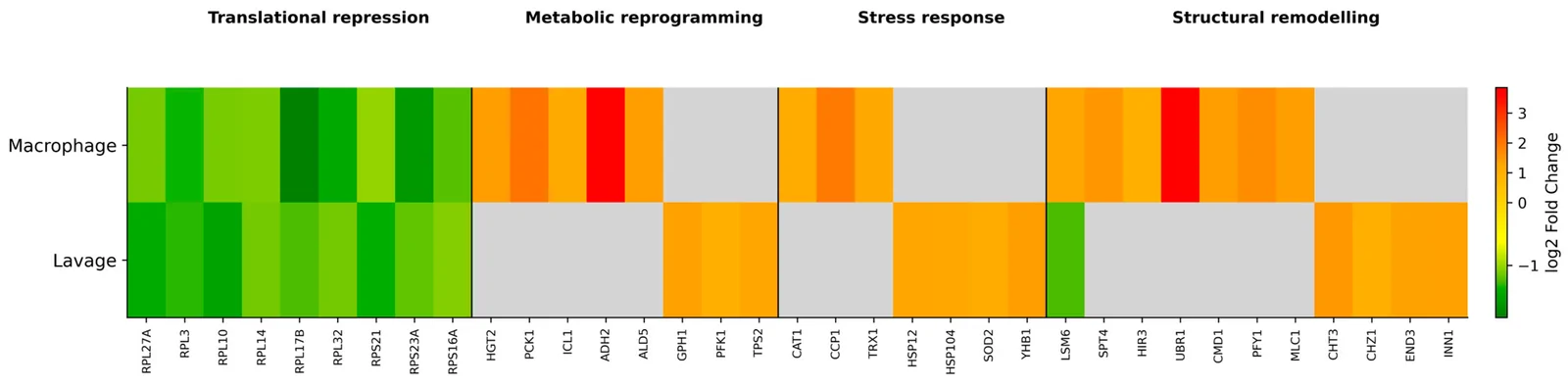
Functional categorization of differentially abundant proteins in macrophage (in vitro) and lavage (in vivo) interaction conditions. Heatmap showing log_2_-fold changes in selected proteins grouped by biological function: translational repression, metabolic reprogramming, stress response, and structural remodelling.

Selected proteins that were differentially abundant were assessed for their potential role in mediating early fungal–pathogen interactions. Functional categories associated with translational control, metabolic processes, stress response, and structural remodelling under both in vitro and in vivo conditions (Figure 5) were determined based on annotations from the Candida Genome Database [19] and manual curation. The results indicated a common downregulation of multiple ribosomal proteins, such as *Rpl27a*, *Rpl3*, *Rpl10*, *Rpl14*, *Rpl17b*, *Rpl32*, *Rps21*, *Rps23a*, and *Rps16a*. Proteins associated with metabolic adaptation were differentially abundant under both environments. During interaction with macrophages in vitro, upregulated proteins included *Hgt2*, *Pck1*, *Icl1*, *Adh2*, and *Ald5*, whereas in vivo lavage samples showed modulation of *Gph1*, *Pfk1*, and *Tps2*. These alterations suggest condition-specific adjustments to utilize carbon. Stress-associated proteins were also upregulated, including *Cat1*, *Ccp1*, *Trx1*, *Sod2*, *Yhb1*, *Hsp12*, and *Hsp104*, consistent with host-associated stress exposure. Finally, proteins linked to structural remodelling were upregulated, including *Cmd1*, *Pfy1*, *Mlc1*, *End3*, *Inn1*, *Cht3*, *Lsm6*, *Spt4*, *Hir3*, and *Ubr1*, indicating proteomic alterations influencing cytoskeletal organization. Altogether, these data suggest a coordinated and context-dependent proteome modulation during the *C. auris* host–pathogen interaction.

## 4. Discussion

To elucidate how *C. auris* mitigates initial host immune responses, we investigated the fungal response during the in vitro interaction with immune cells and in immunocompetent mice. The early-phase infection models used in this study demonstrate that initial *C. auris* survival is influenced by both fungal burden and host context. During the in vitro interaction assay, macrophages significantly reduced fungal viability at an MOI of 1:1; however, no significant killing was observed at an MOI of 10:1, which may indicate macrophage saturation, accentuating the limits of immune clearance. The persistence of *C. auris* at higher fungal densities may also imply the existence of robust immune-evasion strategies, potentially driven by rapid molecular adaptations and collective microbial defence, with the latter reminiscent of biofilm-like conditions. In contrast, following in vivo infection, significantly fewer viable fungal cells were recovered from peritoneal lavage. Although this finding is consistent with fungal clearance in vivo, additional biological and technical factors, including tissue adherence, aggregation, or incomplete lavage recovery, may also contribute to reduced recoverable fungal cells. Therefore, reduced fungal recovery from lavage should not be interpreted exclusively as a direct measure of immune-mediated killing. In order to further delve into the host–pathogen interaction of *C. auris*, a quantitative proteomics methodology was applied to re-isolated fungal cells from the in vitro and in vivo early-phase infection models, which reflected broad proteome remodelling by *C. auris* in response to the host. Of note, it is important to point out the complexities of establishing experimental infection models and their integration thereof; as such, the results of proteomics analysis should serve as elucidations encompassing the early phase of infection and warrant further functional validations. Furthermore, the in vitro and in vivo datasets were generated using different quantitative proteomic workflows; TMT and LFQ, respectively, each with distinct normalization procedures and quantitative characteristics. Consequently, direct comparisons of absolute fold-change magnitudes between datasets should be interpreted accordingly, and conclusions were therefore based primarily on biological inference rather than quantitative cross-platform comparisons. Additionally, the present findings should be interpreted within the context of a single Clade I isolate and a single early infection time point. The observed proteomic responses represent an early molecular snapshot of host adaptation that requires further validation across additional isolates, infection models, and temporal stages of infection.

### 4.1. Translational Repression

A conserved feature of the *C. auris* proteome remodelling during in vitro and in vivo host interactions was the coordinated downregulation of proteins involved in translation and ribosome biogenesis. Multiple ribosomal proteins were downregulated, including *Rpl27a*, *Rpl3*, *Rpl10*, *Rpl14*, *Rpl17b*, *Rpl32*, *Rps21*, *Rps23a*, and *Rps16a* [19,20]. Such a broad repression of translational factors indicates a conserved shift toward a stress-adapted state. Translational attenuation is consistent with cellular stress responses and reflects an adaptive strategy under hostile conditions [21,22]. Such a phenomenon is well documented during *C. auris* interactions with macrophages in vitro [23]. Hence, the shared translational suppression across both in vitro and in vivo environments suggests that exposure to the hostimmune pressure triggers a core adaptive response immediately upon infection. Rather than promoting proliferation, *C. auris* appears to prioritize survival, metabolic efficiency, and stress tolerance.

### 4.2. Metabolic Reprogramming

Immediate exposure to the host environment triggered a wide-spectrum metabolic rewiring by *C. auris*. During macrophage interaction in vitro, enzymes such as high-affinity glucose transporter *Hgt2*, gluconeogenesis enzyme *Pck1*, and glyoxylate cycle enzyme *Icl1* were upregulated, suggesting a shift towards dynamic carbon utilization [24,25,26]. The enzymes alcohol dehydrogenase *Adh2* and aldehyde dehydrogenase *Ald5* were also upregulated, indicating additional involvement of ethanol metabolism [27,28]. The activation of these pathways can be linked to nutrient limitation and intracellular survival and therefore indicates metabolic reprogramming upon macrophage internalization, requiring *C. auris* to rapidly utilize a variety of metabolic substrates. Such an observation is consistent with a recent study where the pathogen’s ability to evade macrophages in vitro was linked to its metabolic adaptation [29]. During in vivo interactions, enzymes such as *Gph1*, *Pfk1*, and *Tps2* were upregulated. The enzyme *Gph1* is essential for glycogen synthesis in *C. albicans* and plays a role in virulence [30]; the pathogen can accumulate carbohydrates in the form of glycogen and mobilize these intracellular reserves when external nutrient availability becomes limited. The upregulation of *Gph1* in vivo suggests that *C. auris* may also engage in a similar adaptive mechanism, whereby host-imposed nutrient constraints may promote glycogen accumulation under nutrient-limited conditions. The enzyme *Pfk1* is a key regulator of glycolysis and has been reported to be a crucial virulence factor in *C. albicans* [31]. The modulation of *Pfk1* in vivo reflects a potential adaptive response by *C. auris* for energy metabolism in response to host-imposed environmental constraints. Finally, the upregulation of *Tps2*, a key enzyme in the trehalose biosynthetic pathway, further supports the notion of adaptive metabolic remodelling by *C. auris* under host-associated stress [32]. Recent work has demonstrated the role of *Tps2* in *C. auris*, including its role in regulating stress tolerance, antifungal susceptibility, and virulence [32]. The enrichment of a wide range of metabolic proteins associated with gluconeogenesis, the glyoxylate cycle, and trehalose metabolism therefore suggests an integrated and rapid metabolic adaptation by *C. auris* upon immediate exposure to host stress. Such a pattern is consistent with metabolic flexibility displayed by numerous pathogens, including *Candida* species, in a host environment where nutrients may be limited.

### 4.3. Stress Response

Hostimmune responses create hostile microenvironments to fend off invading pathogens; in particular, immune cells can generate high levels of reactive oxygen/nitrogen species (ROS/RNS) to impose collective stress [33]. In order to establish a successful infection, pathogens such as *C. auris* must counter such stress responses. Previous investigations have shown the induction of stress-response molecules at the transcriptional level by *C. auris* upon exposure to macrophages in vitro [23]. In agreement with this, under both in vitro and in vivo conditions, an immediate activation of a robust stress response at the proteome level was observed at the early infection phase. During macrophage interaction in vitro, oxidative stress regulators, including *Cat1*, *Ccp1*, and *Trx1*, were upregulated;enzymes that are known to mediate ROS detoxification in *C. albicans* [34,35,36,37,38]. In contrast, in vivo exposure induced a broader stress profile marked by *Hsp12*, *Hsp104*, *Sod2*, and the nitric oxide scavenger *Yhb1*. The proteins *Hsp12* and *Hsp104* have been reported to chaperone virulence and survival in *C. albicans* during host interactions, particularly to mitigate osmotic and thermal stress [39], while *Sod2* mitigates ROS toxicity [38]. The upregulation of *Yhb1* suggests adaptation to host-derived nitric oxide, paralleling nitrosative stress responses described in *C. albicans* host–pathogen interaction [40]. Collectively, induction of these stress mediators underscores a coordinated oxidative and general stress adaptation strategy, enabling *C. auris* to withstand immediate ROS/RNS exposure early on within hostile host microenvironments.

### 4.4. Structural Remodelling

Morphological adaptation emerged as another distinguishing feature during *C. auris* interaction with the host in vitro and in vivo. During in vitro interaction, several molecules mediating structural responses were upregulated, such as *Lsm6*, *Spt4*, *Hir3*, *Ubr1*, *Cmd1*, *Pfy1*, and *Mlc1*. The proteins associated with *Lsm6* and *Spt4* were reported to play a role in filamentation in *C. albicans* [41]. *Hir3* is a component of the HIR complex, which in *C. albicans* has been implicated in regulating morphological plasticity, from yeast to hyphae, which is a key virulence-associated trait [42]. Additional regulators such as *Ubr1* and *Cmd1*, encoding calmodulin, have also been reported to mediate hyphal development in *C. albicans* [43,44]. The upregulation of these yeast-to-hyphae associated factors in our dataset suggests the induction of pseudohyphal growth in *C. auris*, immediately upon macrophage interaction. Morphological transitions have been previously described in *C. auris* under genotoxic stress [45], raising the possibility that immune-mediated stress may induce fungal morphogenesis. Such structural plasticity was further accentuated by the upregulation of the myosin component, *Mlc1*, and profilin *Pfy1*; these were reported to be crucial molecules that modulated fungal growth, cell wall integrity, and virulence in *C. albicans* [46,47]. Altogether, these findings indicate coordinated cytoskeletal and morphogenetic remodelling of *C. auris* during macrophage interaction. During in vivo interactions, proteins such as *Cht3*, *Chz1*, *End3*, and *Inn1* were upregulated. The enzyme *Cht3* is a key chitinase involved in cell wall remodelling and contributes to virulence in *C. albicans* [48,49]. *Chz1* is an essential chitin synthase-associated protein, which has been shown to regulate cell wall integrity [50], whereas *Inn1* has been reported to regulate chitin synthase [51]. As such, both of these proteins can modulate cell morphogenesis by regulating chitin turnover in *C. auris*. Additionally, such chitin-associated molecules have been reported to be downregulated following antifungal exposure [52], suggesting their potential role against stressors. Therefore, the induction of chitin-remodelling proteins in vivo likely contributes to adaptive cell wall remodelling in *C. auris* in response to host-imposed stress.

### 4.5. Potential Moonlighting Function

Moonlighting proteins perform distinct, often unrelated functions depending on cellular localization or microenvironmental context, and are increasingly recognized as important virulence factors in fungal pathogens [53,54]. Several proteins identified in this study may possess additional ‘moonlighting’ functions. For example, the glycolytic enzyme *Pgk1* has been detected on the cell surface where it can bind plasminogen [55], suggesting functions beyond central carbon metabolism. Similarly, pyruvate decarboxylase *Pdc11* has been reported to localize on the cell surface in *C. albicans*, indicating potential non-metabolic roles [56]. The protein *Gna1*, essential for UDP-N-acetylglucosamine synthesis, has also been identified on the cell surface, plus it has also been reported to mediate fungal survival in the host [56,57]. Likewise, *Yke2* has been associated with both carbon metabolism and morphogenesis, thereby priming metabolic function for structural remodelling [58]. Furthermore, *Trx1* has been known for its multifaceted role in promoting fungal survival in the host, in addition to mitigating oxidative stress [34]. The upregulation of such proteins by *C. auris* may therefore reflect not only metabolic engagement but also context-dependent roles that drive early-phase host–pathogen interactions.

## 5. Conclusions

Collectively, our findings suggest that immediate exposure to host environments drives a coordinated and multifaceted adaptive response in *C. auris*. A conserved translational repression across both in vitro and in vivo conditions indicates a potential core adaptive response, perhaps prioritizing survival over proliferation. Apart from the core response, context-dependent divergence in fungal stress and metabolic responses was observed. Interaction with macrophages in vitro was characterized by metabolic reprogramming toward alternative carbon utilization and cytoskeletal remodelling, consistent with intracellular adaptation, whereas in vivo interactions leaned toward carbohydrate metabolism and coordinated cell wall remodelling. At the same time, robust oxidative and nitrosative stress responses underscore the capacity of *C. auris* to withstand host-imposed ROS/RNS pressure. Structural remodelling, through morphogenetic regulation and chitin-associated enzymes, further highlights the adaptive plasticity of *C. auris* during host–pathogen interactions. The identification of moonlighting proteins also suggests that metabolic and stress-associated molecules may additionally contribute to host–pathogen interactions beyond their conventional roles. Altogether, our findings indicate that early adaptation of *C. auris* to host environments is accompanied by the coordinated modulation of translational machinery, metabolic rewiring, stress response, and structural remodelling; a framework warranting further investigation and functional validations. Importantly, these findings provide novel insight into the immediate early-phase molecular adaptations employed by *C. auris* during its interaction with host immune environments, thereby expanding the current understanding of fungal host–pathogen interactions and infection biology.

## Data Availability

The mass spectrometry proteomics data have been deposited in the ProteomeXchange Consortium via the PRIDE partner repository under the dataset identifiers PXD074745 and PXD074762.

## Supplementary Materials

The following supporting information can be downloaded at: https://www.mdpi.com/article/10.3390/jof12090649/s1, Table S1: Supplementary.
